# Resting blood pressure reductions following isometric handgrip exercise training and the impact of age and sex: protocol for a systematic review

**DOI:** 10.1186/s13643-015-0164-6

**Published:** 2015-12-10

**Authors:** Danielle C. Bentley, Cindy H. Nguyen, Scott G. Thomas

**Affiliations:** Faculty of Kinesiology and Physical Education, University of Toronto, 55 Harbord St, Toronto, Ontario M5S 2W6 Canada

**Keywords:** Handgrip exercise, Blood pressure, Aging, Cardiovascular health, Cardiovascular risk

## Abstract

**Background:**

The risk of developing cardiovascular disease is directly correlated to one’s resting blood pressure (BP), age, and biological sex. Resting BP can be reduced using handgrip exercise training, but the impact of age and sex on the effectiveness of training is not well documented.

**Methods/design:**

A systematic search of the literature will be conducted for all experimental studies (including randomized controlled trials and prospective experiments) that report the influence of isometric handgrip exercise training on resting systolic blood pressure. The databases Medline, Embase, Cochrane Reviews, Cumulative Index to Nursing and Allied Health Literature (CINAHL), SPORTDiscus, Web of Science, Allied and Complementary Medicine (AMED), PubMed, and Scopus will be searched until 1 December 2015. Screening of potential articles, data abstraction, and quality appraisal will be completed in duplicate independently. When necessary, corresponding authors will be contacted in order to facilitate the separation of pooled data into age and sex categories. Methodological quality will be determined using the Quality Assessment Framework developed by the Cochrane Collaboration and the Newcastle-Ottawa Quality Assessment Scale as appropriate. Any discrepancies will be resolved by a third author. Findings will be presented in accordance with the preferred reporting items for systematic reviews and meta-analysis (PRISMA) guidelines.

**Discussion:**

This systematic review will determine the overall effectiveness of handgrip exercise training in improving resting blood pressure. A novel, focused assessment will contrast effectiveness of handgrip training based on the age (younger 18–54 years, older >55 years) and the sex (men, women) of study participants. This information is essential to consolidate before moving forward with the development and implementation of handgrip exercise training programmes which are designed to best meet the needs of particular cohorts.

**Systematic review registration:**

PROSPERO CRD42015019792

**Electronic supplementary material:**

The online version of this article (doi:10.1186/s13643-015-0164-6) contains supplementary material, which is available to authorized users.

## Background

### Rationale

Cardiovascular disease (CVD) is the number one cause of death worldwide, representing nearly one third (31 %) of global deaths in 2012 [1]. There is a strong, independent correlation between CVD morbidity and mortality and high blood pressure (BP) [2], with more than 50 % of all CVDs directly related to high BP [1]. Therefore, maintaining BP at an optimal level is critical in order to reduce the global burden of CVD [3].

In addition to the direct impact of high resting BP, the risk of developing CVDs is also influenced by both an individual’s age and their biological sex. Although women typically present with CVD 10 years later than men, a trend that is typically attributed to men presenting with greater risk factors in early age [4], the exponential rise in CVD around the age of 55–60 years in women suggests menopause augments CVD risk [5].

The disparity in CVD incidence between aged men and women is now recognized worldwide, with the American Heart Association publishing separate guidelines for CVD prevention for men and women [6–8] and the European Society of Cardiology formally publishing sex-specific public policy documents [9]. Among Canadian women specifically, the relative risk of developing CVD increases fourfold after the menopause transition [10], a statistic that highlights the impactful interaction of age and biological sex.

To diminish the risk of CVD, it is recommended that individuals with above optimal BP engage in non-pharmaceutical interventions, such as exercise training [11]. Although regular aerobic exercise (i.e. jogging, cycling) consistently reduces resting BP (−3.5/−2.5 mmHg) [12], barriers such as lack of exercise self-efficacy, physical limitations, and financial obstacles can limit uptake. An alternative to traditional aerobic exercise is isometric handgrip (HG) exercise, which is easily accessible, requires little time, and may serve to introduce exercise behaviours to reluctant individuals. The American Heart Association’s most recent (2013) scientific statement on alternative, non-pharmaceutical, approaches to lowering BP gave isometric exercise training a class IIB level of evidence C recommendation, highlighting a need for more research in the field with broader populations [13]. Recently, Carlson and colleagues [14] systematically reviewed the isometric resistive exercise literature, identified nine studies that met rigorous research design criteria (e.g. randomized clinical trial, duration >4 weeks), and reported a significant reduction in resting BP (mean to confidence interval ratio: systolic BP −6.77:−7.93 to −5.62 mmHg; diastolic BP −3.94:−4.73 to −3.16 mmHg). However, the small number of included studies precluded statistical assessments for the potential impacts of age or sex on the outcomes [14]. This limitation has also been encountered among previous literature reviews of isometric exercise which consistently conclude strong overall effectiveness without conducting sub-analyses of age or sex groups [2, 15–17]. Furthermore, many previous reviews include a mixture of training stimuli such as trials of isometric leg extension exercise in addition to isometric HG exercise [12, 14, 17]. A focused review of HG exercise training which assesses the impact of participants’ age and biological sex has yet to be completed.

### Review objectives

A systematic review of isometric HG exercise using a broad set of eligibility criteria and an inclusive search strategy will be undertaken with the primary goal of assessing the potential impacts of participants’ age and sex on the effectiveness of HG exercise training on reducing resting BP.

This review will be the first to identify and segregate the potential influence of participants’ age and sex, as well as the interaction of these two defining categories. We are especially interested in segregating and describing the main effects of training for the specific cohort of post-menopausal women. Together, this information will be instrumental in providing a rationale for the development and implementation of HG training programmes which are designed to best meet the needs of particular cohorts.

## Methods/design

The proposed systematic review conforms to the Preferred Reporting Items for Systematic Review and Meta-Analysis Protocol (Additional file 1) guidelines [16]. In accordance with the PRISMA-P guidelines, this systematic review protocol was registered with the International Prospective Register of Systematic Reviews (PROSPERO) on 23 June 2015 (registration number CRD42015019792).

### Eligibility criteria

#### Types of studies

The inclusion criteria have been designed to be purposefully broad. We will include prospective research designs classified as RCTs, RCTs with a crossover design, experimental exercise interventions without a designated control group, and pilot studies. We will exclude retrospective designs, case series, and case reports.

#### Participants

We will include studies that recruit adult humans ≥18 years of age. We will categorize participants as being “younger” (≤55 years) or “older” (≥55 years). If necessary, authors of studies with participant’s ages across our categories will be contacted directly in order to facilitate segregation of results based on age. It is anticipated that participants may have various comorbidities (i.e. hypertension, heart failure, diabetes) with an assortment of medication use. All comorbidities and medications will be numerically coded accordingly. We will include studies that recruit men, women, or mixed samples and will categorize participants based on biological sex. If necessary, authors of mixed-sex studies will be contacted directly in order to facilitate the segregation of results based on sex.

#### Interventions

Of interest are interventions that use at least 4 weeks of isometric HG exercise as a training modality and measure resting and/or ambulatory systolic BP before and after the training programme. It is anticipated that HG exercise interventions will vary in range of prescribed grip intensities, length of each individual grip contraction, frequency of HG use, etc. All training details will be captured in the data abstraction with the overall training stimulus quantified using a calculation of effort using a time (in seconds) tension (in percent of maximal volitional contraction) product, such that TTP = Time (sec) × Tension (%MVC).

#### Comparators

We anticipate that a small proportion of included studies will use a defined control group. As such, we are planning to use the within study pre- to post-intervention change scores as the assessment of training effectiveness for all cardiovascular variables. Planned sub-analysis comparisons include an analysis of the impact of age (younger versus older) and the impact of sex (women versus men).

#### Outcomes

Eligible studies will examine the impact of HG exercise training on resting systolic BP. Studies which do not assess a pre- to post-intervention change score, or provide the necessary information for the reviewers to extract such information, will be deemed ineligible. Change in BP may be a primary outcome of the included studies or a secondary/tertiary outcome. Included studies may also assess (as a primary, secondary, or tertiary outcome) the impact of HG training on a variety of additional cardiovascular assessments.

#### Timing

Studies will be eligible for inclusion only if the training intervention is at least 4 weeks in duration. A list of excluded publications with interventions less than 4 weeks in duration will be provided as an appendix.

#### Setting

Eligibility will not be restricted to specific exercise settings. Exercise training may occur in a controlled laboratory, at the participant’s home, in a group training environment, or as a combination of settings. In addition, eligibility will not be restricted to level of supervision. Exercise may be completed with an automated digital grip device or a less sophisticated grip device (i.e. spring or elastic band). Both exercise setting and level of supervision will be coded during the data extraction process.

#### Language

We will include articles reported in the English, Portuguese, and French languages. A list of possibly relevant titles in other languages will be provided as an appendix.

### Information sources

We will perform a systematic, computer-assisted, literature search of existing evidence using the online databases of Medline, Embase, Cochrane Reviews, Cumulative Index to Nursing and Allied Health Literature (CINAHL), SPORTDiscus, Web of Science, Allied and Complementary Medicine (AMED), PubMed, and Scopus. To ensure literature saturation, reference lists from both published relevant reviews and retrieved articles will be hand-searched and additional papers assessed for eligibility. We anticipate contacting corresponding researchers to segregate published results for our desired sub-analyses on age (younger versus older) and sex (women versus men).

### Search strategy

No study design, date, or language limits will be imposed on the search strategies. When available, search limits will be used on the variables of AGE (“adult <18–64” and “aged <65+>”) and TYPE (“human”). Relevant studies published up to 1 December 2015 will be retrieved using a specific search strategy created in conjunction with a University of Toronto research librarian with expertise in systematic reviews. The search strategy was kept purposefully broad to increase the opportunity to identify potentially relevant papers. A representative OVID keyword search transcript is presented here which will be applied to the databases of Medline, Embase, and AMED.

1. [handgrip] OR [isometric grip] OR [static grip] OR [forearm grip]
2. [training] OR [intervention*] OR [exercise*] OR [physical activity]
3. [blood pressure] OR [systolic] OR [cardiovascular]
4. [1] AND [2] AND [3]

### Study records

#### Data management

Titles and abstracts from identified articles will be imported into EndNote (version 5.0 Thompson Reuters, 2011), an electronic reference management software. Extracted data from eligible studies will be entered in to a custom-made abstraction framework in Microsoft Excel (version 12.3.6, Microsoft Corporation, 2007) and analyzed using SPSS (version 22, IBM SPSS Statistics, 2015).

#### Selection process

Following duplicate removal, the entire list of identified articles will be independently screened by DB and CN, with discrepancies assessed for a third time by ST. Overall, the screening process will include titles and abstracts of potentially relevant articles to appraise eligibility using the custom-made screening form. Eligible articles will be those that study the direct impact of ≥4 weeks of isometric HG exercise training on measures of cardiovascular health within a cohort of adult humans. Those articles which do not meet the eligibility criteria will be excluded. Reference lists of retrieved articles and published relevant reviews will be hand-searched for additional papers and assessed for eligibility. Full texts of all studies meeting the inclusion criteria will be retrieved and printed for detailed review and data abstraction using a custom-made abstraction framework.

#### Data collection process

Data abstraction from all included articles will be independently completed by DB and CN with discrepancies assessed by ST. A custom-made data abstraction framework will be used to record significant study characteristics. Amendments to the abstraction framework and corresponding data extraction variables will be made upon review of included articles, if necessary. When required, corresponding authors will be contacted directly to obtain necessary additional information, such as data separation for age and sex assessments. It is possible that the same data from a single study may be presented in multiple reports. In order to avoid this, studies with at least one shared author will have participants’ details directly compared to assess potential duplication of results.

### Data items

It is anticipated that a diverse range of research methodology will be identified within this review, and hence, a statistical meta-analysis is unlikely to be appropriate. We will extract information regarding study design details (i.e. year of publication, exercise prescription, bilateral or unilateral exercise, time-tension product of HG protocol); participants’ details (i.e. age (segregate for younger and older), sex (segregate for male and female), resting BP status (normotensive (<120/<80 mmHg), above optimal (>121/>81 mmHg)), medication use, comorbidities); primary cardiovascular variables (i.e. impact of HG training on blood pressure, heart rate, arterial health and function measurements, venous health and function measurements); and any exercise training adherence information (i.e. number of drop-outs, compliance to exercise protocol). Location of exercise (primarily at home, primarily in the laboratory, etc), level of supervision (digital grip output/in-laboratory supervision, grip device without feedback/unsupervised), method of BP assessment (resting, ambulatory), and timing of final assessment compared to final exercise bout (<24 hours (to avoid overlapping results with post-exercise hypotension), >72 hours (to avoid effects of detraining), and 24–72 hours (ideal)) will be coded.

We will make the assumption that women in our “older” age category (≥55 years) will be post-menopausal. Although natural menopause in women can be affected by a variety of factors such as ethnicity, diet, physical activity, and genetics [18], the National Institute of Aging states that the most recent average age of menopause is 51 [19].

### Outcomes and prioritization

Our primary outcome of interest will be the following:

- Change in systolic BP as a result of isometric HG exercise training. Systolic BP can be measured as resting or ambulatory. Studies which do not report change in systolic BP over time will be excluded.

Secondary outcomes of interest will be collected and analyzed if sufficiently reported. They will be as follows:

- Change in resting diastolic BP
- Change in heart rate
- Change in arterial health and function (i.e. pulse wave velocity, arterial distensibility, reactive hyperaemic forearm blood flow, flow mediated vasodilation)
- Change in venous health and function (i.e. venous compliance)
- Change in autonomic nervous system indicators (i.e. muscle sympathetic nerve activity, heart rate variability, blood pressure variability, and cardiovascular reactivity)

For all included outcomes, we will segregate out the impact of age (younger versus older) and sex (women versus men).

### Assessment of study quality

It is anticipated that included studies will be of various research designs, some with and some without a randomized control group. Therefore, we will utilize two corresponding assessment tools. For randomized controlled trials we will use the Quality Assessment Framework developed by the Cochrane Collaboration to assess the following sources of bias: selection bias (random sequence generation and allocation concealment), performance bias (blinding of participants/personnel and other potential threats to validity), attrition bias (incomplete outcome data), detection bias (blinding of outcome assessment), and reporting bias (selective outcome reporting) [20]. For experimental exercise interventions without a designated control group, we will use an adapted form of the Newcastle-Ottawa Scale (NOS) [21]. Both the Quality Assessment Framework and the NOS will be adjusted for realistic allocation of points for blinding. With exercise training studies, it is seldom feasible for research participants to be blinded to their intervention group. However, blinding of researchers completing data collection and blinding of participants to the primary outcome variable of interest are still reasonable.

### Data synthesis

#### Minimum criteria

Before proceeding with statistical analysis of results, studies which have been identified as having a “high risk of bias” using the aforementioned Cochrane Collaboration risk of bias assessment tool or having less than four stars on the NOS star rating will be removed.

#### Planned summary of measures, exploration of consistency, and planned subgroup analyses

A narrative summary of the included studies will be presented. This will include a summary of the study design, participants’ details, descriptive cardiovascular variables, and any exercise training adherence information. Reported changes to our primary and secondary outcomes (i.e. blood pressure, heart rate, vascular health assessments) will be calculated as weighted mean differences (with a 95 % CI). When available, clinical cardiovascular measures will be segregated based on the age and the sex of individual participants. We anticipate that systematic differences between studies will be likely, and we will assess both the presence (chi-square test) and impact (*I*^2^ and its confidence interval) of this heterogeneity. The data will be considered too heterogeneous to pool if the confidence interval around *I*^2^ does not contain the 0 % value [22]. If data are too heterogeneous to pool, or are not reported in a format suitable for pooling (i.e. data reported as medians), then we will complete a narrative synthesis of the presented data. We will use both descriptive text and tables to summarize the data in a way that encourages the reader to consider the clinical outcomes in light of difference in study designs. Studies will be organized (if appropriate) by study design details, participants’ details, descriptive cardiovascular variables, and any exercise training adherence information. A detailed commentary on the major methodological problems or biases that affected the studies (if any) will also be included, together with a description of how this has affected the individual study results. Throughout the planned review, planned subgroup data presentation will explore (1) participants’ characteristics such as age, sex, and resting BP status at commencement of exercise training; (2) exercise characteristics such as HG force prescription, length of training, and type of tool used; and (3) location of exercise training such as at-home or in-laboratory.

#### Planned synthesis presentation

The presentation of review results will be constructed in accordance with the PRISMA guidelines.

### Strength of the evidence

The Grading of Recommendations, Assessments, Development and Evaluation (GRADE) approach will be used to assess the quality of evidences for all review outcomes across the domains of risk of bias, inconsistency, imprecision, indirectness, publication bias, and factors that increase the confidence in an effect (i.e. large effect sizes, dose-effect relations).

## Discussion

### Strengths and limitations

A strength of this review is the systematic and transparent approach that is being taken, which draws on recommended and validated methods [18]. The review is thorough and broad, ensuring a comprehensive representation of information results. In addition, the use of two separate reviewers for the screening process, data abstraction, and quality appraisal will increase the strength of conclusions.

A potential limitation of this review is the anticipated volume of literature. Although HG exercise has been used for decades as a short-term stressor, the use of HG as a training modality to reduce blood pressure is more recent. Therefore, it is anticipated that the majority of published research using HG exercise training will have been conducted in the last 5 years due to the focused attention of isolated research groups. As such, this review will include a description of included articles, such as a number of articles published each year, to enhance transparency.

In conclusion, we will perform this systematic review to determine the overall effectiveness of HG exercise training in reducing resting BP. A focused assessment will contrast effectiveness in men compared to women and younger compared to older study participants. This information is essential to consolidate before moving forward with the development and implementation of HG or alternative exercise training programmes which are designed to best meet the needs of particular cohort, such as aged women. In the immediate future, the findings from this review will be useful for exercise researchers and clinicians. It is anticipated that following supplementary primary research, this information will be useful for individuals looking to manage their BP through non-pharmaceutical interventions.

### Current stage of systematic review

PROSPERO (stage 3). Formal Screening of search results has just commenced. With 1238 hits, the screening will take a significant period of time to complete.

## Additional file

Additional file 1:
**The PRISMA-P checklist has been completed and uploaded as per required by the Systematic Reviews author guidelines.** (PDF 146 KB)

## Abbreviations

BP: blood pressure
CVD: cardiovascular disease
GRADE: Grading of Recommendations, Assessments, Development and Evaluation
HG: handgrip
MVC: maximal volitional contraction
NOS: Newcastle-Ottawa Scale
PRISMA-P: Preferred Reporting Items for Systematic Review and Meta-Analysis Protocol
PROSPERO: Prospective Register of Systematic Reviews
RCT: randomized control trial
TTP: time-tension product
